# Prospective cohort study on recovery outcomes in elderly hip fracture patients using minimally invasive techniques

**DOI:** 10.6026/973206300211616

**Published:** 2025-06-30

**Authors:** Priyanka Perumal, Harsh C. Shingala, Shreyas Madagundanahalli Srinivasa, Rakshana Munusamy, Ajeet Saoji, Naman Dimpal Shah, Goutham Murugesan, Shoraf P.

**Affiliations:** 1 Department of General Surgery, SRM Medical College Hospital and Research Centre, Chengalpattu, Tamil Nadu, India; 2 Department of Medicine, HCG Hospitals, Ahmedabad, Gujarat, India; 3 Department of Trauma & Orthopedics, Apollo Hospitals, Bengaluru, Karnataka, India; 4 Department of Surgery, Madurai Medical College, Chennai, Tamil Nadu, India; 5 Department of Community Medicine, N. K. P. Salve Institute of Medical Sciences & Research Centre And Lata Mangeshkar Hospital, Nagpur 440019, Maharashtra, India; 6 Department of Medicine, Orient House Medical Centre, Bolton, UK; 7 Department of Intensive Care, Orthomed Hospital, Royapettah, Chennai, Tamil Nadu, India; 8 Department of Community Medicine, Madha Medical College & Research Institute, Chennai, Tamil Nadu 600128, India

**Keywords:** Hip fractures, elderly, minimally invasive surgery, functional recovery, complication rates, hospital stay, orthopedics

## Abstract

Hip fractures in elderly patients are most often associated with a high degree of morbidity and mortality. The effectiveness of early intervention cannot be overemphasized. A prospective cohort study on elderly patients treated with minimally invasive surgical techniques to recover discusses issues on functional recovery, complications and length of hospital stay. Data were collected from 120 patients aged 65 years and above for a period of 12 months. The findings of the study indicated that minimally invasive techniques were associated with improved functional recovery and fewer complications than traditional methods, making them valuable in the care of elderly patients. Such results support the wider use of minimally invasive techniques in managing hip fractures among older populations.

## Background:

Hip fractures form a major clinical concern among older adults, following low-energy fall due to loss of bone and other changes accompanying aging [1]. Substantial morbidity and mortality exist with these conditions, coupled with long-term disabilities that seriously threaten the quality of life [2]. These injuries need a timely surgical procedure to regain mobilization, ward off complications and reduce the rising costs of medical care [3]. Traditional open surgical techniques for the treatment of hip fractures are associated with significant morbidity, such as increased risks of infection and blood loss and prolonged recovery periods [4]. Minimally invasive techniques are emerging as an alternative option with benefits like less surgical trauma, fewer complications and more rapid rehabilitation [5]. These techniques intend to improve the functional recovery and minimize postoperative risks by preserving soft tissues and avoiding disruption at the fracture site [6]. Even though there is an increased adoption of these minimally invasive methods, few data are available regarding their benefits in elderly populations who often have multiple comorbidities and decreased physiological reserve [7]. Therefore, the outcomes in this population concerning such techniques still remain under evaluated; with respect to function recovery rates of complications and length of hospital stay [8]. These factors, therefore, provide a basis in optimizing surgical techniques and improving results for patients. This prospective cohort study assesses recovery outcomes after minimally invasive surgical fixation in the elderly hip fracture population. Through comparison of functional recovery, complications and hospital stay duration this article offers evidence to be used to demonstrate the potential benefits of the minimally invasive approach in care of the elderly. The discovery would be an addition to guidelines on clinical decisions that could evolve the standard of protocols for management of hip fractures among the older age group.

## Materials and Methods:

This is a prospective cohort study conducted for 12 months at a tertiary hospital in order to evaluate the outcomes of recovery of elderly patients suffering from hip fractures with minimally invasive surgical techniques. A total of 120 patients were recruited with a minimum age of 65 years and radiologically confirmed hip fractures were included. Patients with pathological fractures or prior hip surgeries and severe systemic conditions contraindicating surgery were excluded. Baseline data of demographics, comorbid conditions and types of fracture were collected. The surgical methods applied were either percutaneous screw fixation or intramedullary nailing based on the type of fracture and surgeon discretion. Preoperative optimization was through improvement of comorbid conditions, anticoagulation management and nutrition. Early mobilization, pain control and physiotherapy are considered postoperative care. Recovery outcomes were evaluated over six months with regard to functional recovery (Harris Hip Score), postoperative complications, length of hospital stay and time to independent ambulation. Ethical approval was obtained from the institutional ethics committee and informed consent was secured. Data analysis used descriptive and comparative statistics to evaluate key outcomes.

## Results:

Table 1 (see PDF) outlines the demographic and baseline characteristics of the study population. The majority of patients were aged 70-79 years and comorbidities such as hypertension and diabetes were common, reflecting the typical profile of elderly hip fracture patients. Table 2 (see PDF) shows functional recovery outcomes based on the Harris Hip Score (HHS) at baseline, 3 months and 6 months post-surgery. Patients demonstrated significant improvement in functional scores over time, indicating the efficacy of minimally invasive techniques. Table 3 (see PDF) highlights the incidence of postoperative complications. The overall complication rate was low, with no significant adverse events requiring major interventions. Table 4 (see PDF) summarizes the length of hospital stay. Most patients were discharged within 5-7 days, indicating the efficiency of minimally invasive techniques. Table 5 presents the time to independent ambulation post-surgery. The majority of patients regained independent mobility within 6 weeks. Table 6 (see PDF) details overall patient satisfaction scores. A high satisfaction rate was observed, reflecting positive perceptions of the minimally invasive approach. The study showed that this minimally invasive surgical procedure on aged hip fracture patients was effective in terms of functional recovery, few complications and a good outcome for patients. Baseline characteristic (Table 1 - see PDF) showed that there were mostly intertrochanteric fractures that comprised 53.3% and that the common co-existing diseases that were noted were hypertension 60% and osteoporosis 74.2%. Functional recovery results (Table 2 - see PDF) were seen to have an excellent improvement in Harris Hip Scores, with a 90.9% increase at six months after surgery. The overall rate of postoperative complications was 10%, which consisted mainly of surgical site infections at 5% and thromboembolic events at 3.3% (Table 3 - see PDF). The majority of the patients were discharged within 5-7 days 60%, (Table 4 - see PDF) and the majority of them regained independent ambulation within 6 weeks 53.3%, (Table 5 - see PDF). Patient satisfaction was extremely high: 73.3% of them were very satisfied with the outcome of surgery in relation to fracture healing (Table 6 - see PDF). This result establishes clinical and functional advantages of using minimally invasive techniques in treating elderly hip fractures.

## Discussion:

This study shows that minimally invasive surgical techniques in elderly hip fracture patients have several benefits, such as faster functional recovery, lower complication rates and shorter hospital stays [9]. Functional results were significantly improved, with an increase of 90.9% in Harris Hip Scores by six months post-surgery and complications were minimal, at 10% overall [10]. Most patients regained independent ambulation within six weeks, showing the effectiveness of minimally invasive methods in encouraging early mobility [11, 12]. There was significant patient satisfaction at 73.3% very satisfied, reinforcing clinical benefits and the reduction of burden linked with these techniques [13]. The study does support minimally invasive approaches as standard care for hip fractures however; multi-center studies with extended follow-up are required to validate long-term outcomes in terms of cost-effectiveness [14, 15].

## Conclusion:

Minimally invasive surgical techniques improve the outcomes of elderly patients with hip fractures significantly through enhancement of functional recovery, decreased complication rates and shortening of hospital stays. Such approaches present patient-centered and efficient solutions to manage hip fractures in older populations. Future studies should focus on validation of long-term benefits and cost-effectiveness for further reinforcement in adoption as a standard of care.
